# Non-cleavable talin rescues defect in the T-cell conjugation of T-cells deficient in the immune adaptor SKAP1

**DOI:** 10.1016/j.imlet.2016.02.004

**Published:** 2016-04

**Authors:** Daina Lim, Yuning Lu, Christopher E. Rudd

**Affiliations:** a Cell Signalling Section, Division of Immunology, Department of Pathology, Tennis Court Road, University of Cambridge, Cambridge CB2 1QP, UK; b Cambridge Institute of Medical Research, Hills Road, CB2 OXY Cambridge, UK

**Keywords:** T-cells, Talin, LFA-1, Adhesion

## Abstract

While the cytoskeletal protein talin binds to the β-chain of LFA-1, the immune cell adaptor SKAP1 (SKAP-55) binds to the α-chain of the same integrin via RapL. Whereas calpain protease cleavage of talin is important for LFA-1 activation, it has been unclear whether SKAP1 can alter the function of talin or its associated adaptor RIAM in T-cells. In this paper, we report that *Skap1−/−* T-cells showed a reduction in the translocation of talin and RIAM to the contact interface of T-cells with antigenic beads or dendritic cells (DCs) presenting OVA peptide to OT-1 T-cells. In addition, *Skap1*−*/*−** T-cells show an altered pattern of talin cleavage, while the expression of a cleavage resistant form of talin (L432G) restored the impaired adhesion of OT1 transgenic *Skap1*−*/*−** T-cells with DCs. SKAP1 therefore can affect the function of talin in T-cells needed for optimal T-cell/DC conjugation.

## Highlights

• *Skap1*−*/*−** T-cells show impaired talin and RIAM localization at the anti-CD3 beads.
• Talin cleavage is altered in *Skap1−/−* T-cells.
• Cleavage resistant talin (L432G) restored normal conjugation of *Skap1−/−* T-cells.
• Immune cell adaptor SKAP1 interfaces with regulation of talin and RIAM in T-cells.

## 1 Introduction

Integrins play central roles in mediating trans-endothelial migration of T-cells and their contact with antigen presenting cells (APCs) [1], [2], [3]. T-lymphocytes express mainly β1, β2 and β7 integrins where LFA-1 comprised of a αL and a β2-chain [2]. LFA-1 is activated by conformational changes for enhanced affinity, and by clustering or avidity changes of the receptor [4], [5]. Chemokine or T-cell receptor (TCR) mediated ‘inside out’ signaling can convert the inactive LFA-1 to either an intermediate and high affinity forms by exposing the intercellular adhesion molecule 1 (ICAM-1)-binding I domain [6]. Increased LFA-1 clustering also increases multivalent binding [6], [7].

The activation of LFA-1 by antigen-receptor ligation depends on many upstream and downstream signaling events such as CD4/CD8-p56^lck^ [8], interleukin 2 (IL-2)-inducible T-cell kinase (ITK) [9], the guanine nucleotide exchange factor Vav-1 [10], [11], phosphatidylinositol 3-kinase (PI 3K) [12], [13] and Rho–Rac GTP binding proteins [11], [14]. Adaptors ADAP (adhesion and degranulation–promoting adaptor protein) (HUGO official designation: Fyb) [15], [16], [17] and SKAP1 (src kinase associated phosphoprotein 1: HUGO official designation; also SKAP-55, src kinase-associated phosphoprotein-55) are also needed for β1 and β2 integrin activation [18], [19], [20]. In fact, we have shown that SKAP1 is an effector is the inside-out pathway by interacting with RapL (regulator of cell adhesion and polarisation enriched in lymphoid tissues) in T-cells [18], [19], [21], [22], [23], [24]. Rap1 [25], [26], [27], [28] also binds RapL [29], [30] and RIAM (Rap1-interacting adaptor molecule) [31].

Talin is a ubiquitous high-molecular-weight protein of the cytoskeleton that is essential for the activation of integrins [32], [33]. It consists of a C-terminal rod domain, a N-terminal FERM domain and multiple alpha helices [34], [35]. The F3 subdomain of the FERM domain binds to the integrin β integrin and is needed to activate integrins [36]. Talin also links the cytoplasmic domain of integrin β-chains to actin filaments and is localised in contact regions between T-cell and antigen-presenting cells such as cytolytic T-cells and their targets [37], [38], [39], [40]. Talin also couples integrins to the actin cytoskeleton by interacting with vinculin and alpha-actinin [41], [42]. Further, it is a substrate for the calcium activated protease, calpain II [43]. Quantification of adhesion assembly and disassembly rates has demonstrated that this proteolysis is a rate-limiting step for adhesion turnover [44]. In this context, disassembly of adhesion components such as paxillin, vinculin and zyxin, is dependent on this cleavage event [44]. Calpain cleavage also promotes talin binding to the β3 integrin cytoplasmic domain and clustering [45]. Talin is targeted to the plasma membrane via the shuttle protein Rap1-GTP-interacting adaptor molecule (RIAM), a member of the MRL (Mig-10/RIAM/Lamellipodin) protein family [46].

Given the binding of talin to the β-chain and the SKAP1-RapL-Rap1 complex to the α-chain in LFA-1, it has been unclear whether these mediators can affect each other. In this study, we report that T-cells from *Skap1*−*/*−** mice show altered processing and localization of talin in T-cells, concurrent with reduced dwell times with DCs, and further that a cleavage resistant L432G talin rescued impaired *Skap1*−*/*−** T-cell conjugation. This observation finding demonstrates cross-regulation between SKAP1 and talin in T-cells despite binding to distinct chains of LFA-1.

## 2 Methods and materials

### 2.1 Reagents

The generation of SKAP1 knock-out mice had been previously described elsewhere [18]. Dynabeads M-450 Epoxy were purchased from Invitrogen (Oslo, Norway). Antibodies against talin (Clone 8D4) was purchased from Sigma–Aldrich (Missouri, USA); anti-RIAM from Protein Tech Group (IL, USA); anti-CD3ε (2C11; hamster-anti-mouse CD3) from Pharmingen (Oxford, UK); anti CD3ε (OKT3, mouse-anti-human CD3) from ATCC. KIM-127 was a kind gift from the lab of Dr. Nancy Hogg (Cancer Research UK). Secondary antibodies—anti-mouse Alexa568 and anti-rabbit Alexa488 were purchased from Invitrogen. GFP-Talin-L432G was a gift from Anna Huttenlocher (Medical Microbiology & Immunology, University of Wisconsin–Madison, US) (Addgene plasmid # 26725).

### 2.2 T-cell isolation

Spleens isolated from C57Bl6 or SKAP1-deficient mice were meshed through cell strainers, followed by removal of red blood cells (RBC) with hypotonic buffer (0.15 M NH_4_Cl, 1 mM NaHCO_3_, 0.1 mM EDTA, pH 7.25). CD3^+^ T-cells were purified from the splenocytes using a Mouse T cell Enrichment column (R&D Systems). Cells were then used immediately for experiments. Primary naïve mouse cells were transfected with various vectors using the Amaxa Nucleofector Kit (Lonza, Germany). Jurkat T-cells were transfected by microporation (Digital Bio Technology) using a single pulse of 30 ms at 1410 V. In certain experiments, mouse and Jurkat T-cells were stimulated with 2–5 μg/ml of 145-2C11 or OKT3, respectively [47].

### 2.3 T-cell conjugation and motility assay

T-cell conjugation and motility assay were conducted as described [48], [49]. *Skap1*−*/−* mice were crossed with OT-1 transgenic mice to generate *Skap1*−*/*−** OT-1 (SKOT1) mice. *Skap1+/+* OT-1 (OT1) vs*. Skap1*−*/*−** OT-1 (SKOT1) T-cells were activated for 3 days with 10 μg/ml OVA peptide, washed and rested for 24 h before use.

### 2.4 Immunofluorescence staining

Immunofluorescence staining was conducted as described. Anti-CD3 coated beads were prepared by incubating 4 μg of anti-CD3ε (2C11) with 10^6^ Dynabeads M-450 Epoxy beads in phosphate buffer for 30 min at 4 °C prior to supplementing with FBS to a final concentration and a further incubation of 0.3% overnight. Alternately, T-cells were plated on polylysine-coated coverslips incubated with anti-CD3 (2 μg/ml) for the stipulated time points. The cells were then washed with PBS to remove any non-adherent cells before fixing in Cytofix (BD Biosciences, Oxford, UK). Cells were then permeabilised using 0.5% Saponin before staining with the relevant antibodies. Anti-mouse Alexa568, anti-rabbit Alexa488, anti-rabbit Alexa647 and anti-mouse Alexa568 were used as appropriate secondary antibodies.

### 2.5 Immunoprecipitation and western blotting

Membranes of cells were isolated from detergent solubilisation for immunoprecipitation. Cells were centrifuged at 1850 rpm for 5 min and wash with PBS before resuspending in cold hypotonic buffer (10 mM HEPES, 1.5 mM MgCl_2_, 10 mM KCl, 0.5 mM PMSF, 5 mM DTT, 0.1 mM NaV) supplemented with protease inhibitors (Roche) for 10 min at 4 °C. Cells were then homogenised before centrifugation at 3300 rpm for 15 min at 4 °C. The pellet is discarded and supernatant is centrifuged at 15000 rpm for 1 h to separate cytosolic fraction from membranes. The cytosolic fraction is collected from the supernatant and the membrane fraction is solubilised with RIPA buffer (50 mM Tris–HCl pH 8.0, 150 mM NaCl, 1% NP-40, 0.5% sodium deoxycholate and 0.1% SDS). Immunoprecipitation and Western blotting was conducted as described [23], [50].

### 2.6 Statistical analysis

Results are given as the mean ± standard deviation (SD). Statistical significance was tested using unpaired student’s *T*-test using GraphPad Prism version 3.02 (GraphPad Software, San Diego, California, U.S.A.), with *p* < 0.05 was considered as significant.

## 3 Results and discussion

### 3.1 SKAP1 is needed for optimal contact times with DCs

**Fig. 1:**
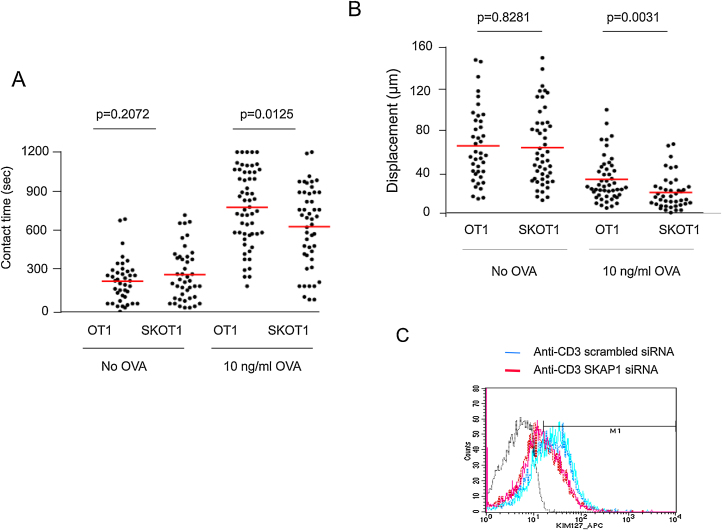
SKAP1 is crucial for the optimal T cell/APC dwell times in OT1 T-cells. CTLs from *Skap1−/−* OT-1 (SKOT1) or *Skap1+/+* OT-1 (OT1) were generated from splenocytes stimulated with OVA peptide for 3 days before rested for 24 h. CTLs labeled with SNARF-1 were seeded with DCs alone or DC-OVA. (A) SKOT1 and OT1CTLs had comparable contact time in the absence of OVA (no OVA) (*p* = 0.2072) (>200 conjugates/test condition/experiment; *n* = 3). (B) SKOT1 or OT1CTLs had comparable displacement with no OVA (*p* = 0.8281) but SKOT1CTLs showed a significant decrease in displacement as compared to OT1CTLs with DC-OVA (*p* = 0.0031). (C) Jurkat T-cells transfected with SKAP1 siRNA show reduced staining with anti-intermediate affinity LFA- MAb KIM-127.

We previously reported that SKAP1 was needed for LFA-1 adhesion and T-cell conjugation in response to super-antigen [18], [20], [23]. To assess the role of SKAP1 in an antigen-specific model, *Skap1*−*/−* OT-1 (SKOT1) mice were used. *Skap1+/+* OT-1 (OT1) vs*. Skap1*−*/*−** OT-1 (SKOT1) T-cells were activated for 3 days with 10 μg/ml OVA peptide, washed and rested for 24 h followed by a measure of dwell times with DCs and motility (Fig. 1A). Mature DCs were prepared as described previously by labeling with SNARF-1 and pre-incubating with OVA_257–264_ peptide (DC-OVA) prior to incubation, as described [51]. The presence of OVA peptide increased contact times from a mean of 237–788 s for OT1 T-cells (*p* < 0.0001). SKOT1 T-cells also showed an increase in contact time from 287 s to 645 s (*p* < 0.0001) in the presence of OVA peptide. However, the mean contact time between SKOT1 T-cells and DCs was significantly lower than between OT1 T-cells and DCs (*p* = 0.0125). The decreased contact time between SKOT1 T-cells and DCs was also accompanied by a decrease in displacement in SKOT1 T-cells (Fig. 1B). To assess an effect of SKAP1 on LFA-1 affinity, we transfected Jurkat T-cells with scrambled vs. SKAP1 siRNA, ligated with anti-CD3 for 30 min and stained cells with the MAb KIM-127 (Fig. 1C). KIM-127 recognizes the intermediate affinity form of human LFA-1 (CD11a/CD18) [52]. Activated human Jurkat T-cells expressing SKAP1 siRNA showed a lower staining than cells expressing scrambled siRNA (Fig. 1C). Overall, these data demonstrated that SKAP1 is needed for LFA-1 activation and contact between T-cells and DCs.

### 3.2 SKAP1 is required for the optimal translocation of talin and RIAM and elongation of T-cell in response to TCR activation

**Fig. 2:**
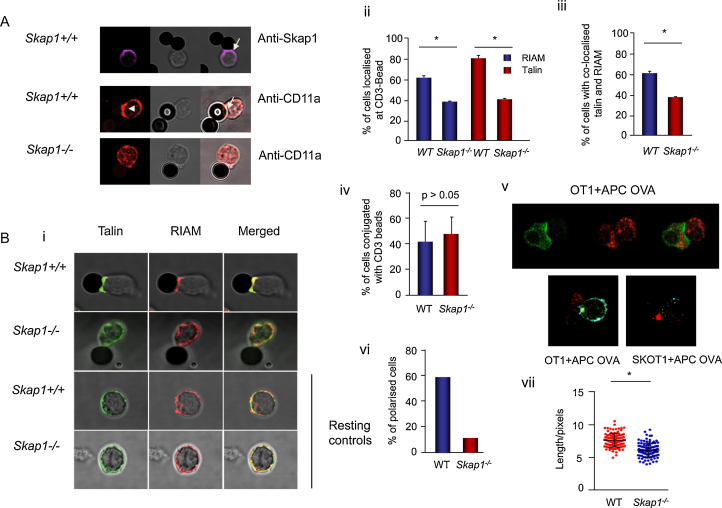
SKAP1 regulates the translocation of talin and RIAM to the immunological synapse and elongation of the T-cells upon TCR stimulation. (A) LFA-1 translocation at the immunological synapse (IS) upon stimulation of TCR is dependent on SKAP1. Naïve CD3^+^ T-cells incubated with anti-CD3 coated beads for 1 h were stained with anti-SKAP1 or anti-CD11a plus Alexa-Fluor 568 were imaged using confocal microscopy. SKAP1 and CD11a were found to be present at the bead contact site for *Skap1+/+* T-cells. CD11a was found to be diffusely located in *Skap1−/*−** T-cells. (B) Translocation of talin and RIAM to IS upon TCR stimulation is dependent on SKAP1. (i) Representative images of talin and RIAM distribution in *Skap1+/+* and *Skap1*−*/−* naïve T-cells in contact with anti-CD3 beads. Naïve T-cells were incubated with anti-CD3 coated beads for 1 h were fixed and stained with anti-RIAM plus Alexa-Fluor 568 followed by anti-talin plus Alexa Fluor 468, and were imaged using confocal microscopy. (ii) Histogram shows the percentage of naïve T- cells with RIAM or talin localised at the bead contact site (*n* = 3, 120–160/experiment). The means ± SD are displayed. (iii) Histogram shows the percentage of naïve T-cells with RIAM and talin co-localised at the bead contact site (*n* = 3, 120–160/experiment). (iv) Histogram shows the percentage of naïve T-cells in conjugation with an anti-CD3 bead with RIAM and talin co-localised at the bead contact site (*n* = 3, 120–160/experiment). (v) CTLs from SKOT1 or OT1 show similar distribution of talin and RIAM when in contact with an APC previously incubated with OVA peptide. CTLs from SKOT1 or OT1 were generated from splenocytes with OVA peptide for 3 days before rested for 24 h before incubating with DC-OVA for 10 min. Cells were fixed and stained with anti-talin plus Alexa Fluor 468 (green) and were imaged using confocal microscopy. (vi) Histogram shows the percentage of polarised cells. A polarised cell was defined as at least 1.5 times the mean diameter of an unstimulated cell (*n* = 3, 120–160/experiment). (vii) Histogram shows the length of cells, measured lengthwise from the point of anti-CD3 bead contact site (*n* = 3, 120–160/experiment). The means ± SD are displayed. Differences between means are compared using two-tailed unpaired Student’s *T*-test (*, *p* < 0.05).

While SKAP1 regulates the Rap1-RapL binding to the αL chain of the LFA-1 [23], talin binds to the β-subunit and is needed for LFA-1 activation [53], [54]. We initially asked whether SKAP1 might affect talin and RIAM localization with LFA-1 at an activation interface (Fig. 2). To assess this, beads coated with anti-CD3 were initially used. They were incubated with primary naïve T-cells from WT and *Skap1*−*/−* mice followed by staining of talin and its associated protein RIAM by confocal immunofluorescence. Anti-CD3 induced the translocation of SKAP1 to the contact site with beads as seen in wild-type cells (Fig. 2A, upper panels). Concurrent with this, the clustering of LFA-1 was observed at the contact with beads in *Skap1+/+* T-cells and this was reduced in *Skap1*−*/−* T-cells (Fig. 2A, middle and lower panels), as reported [23], [24].

We next looked at the translocation of talin and its associated adaptor RIAM to the contact site (Fig. 2B). With *Skap1+/+* T-cells, 81% of the cells showed translocation of talin to the beads that was significantly reduced to 42% with *Skap1*−*/−* T-cells (Fig. 2Bi upper panel and ii). A similar difference was noted with the localization of RIAM where 61% of *Skap1+/+* T-cells showed RIAM localization compared to 38% for *Skap1*−*/*−** T-cells (*p* < 0.005) (Fig. 2Bii). Further, most of the RIAM was also found outer contact region with beads (Fig. 2Bi upper panel) and was co-localized with talin (Fig. 2Biii). By contrast, no difference in the number of *Skap1+/+* vs. *Skap1*−*/−* T-cells was found associated with the anti-CD3 beads (Fig. 2Biv). A similar situation was observed between OT-1 T-cells (CTLs) with peptide presenting EL4 cells with localized cap of talin at the contact site of Skap1+/+ T-cells (Fig. 2Bv**,** left panel, see arrow), with was diffused in *Skap1*−*/*−** (SKOT1) cells (right panel). These findings showed that anti-CD3 on beads or OVA peptide presentation b induction of the translocation of talin to the contact point requires the expression of SKAP1. This differences of IS localization correlated with the reduced polarisation of *skap1*−*/*−** T-cells at the interface of anti-CD3 coated beads (Fig. 2Bvi). Polarization of T-cells was defined as 1.5 times the mean diameter (Fig. 2Bvi). 58% of the WT T-cells were polarized relative to 9% of *Skap1*−*/*−** T-cells. *Skap1*−*/*−** T-cells also showed less length extension when in contact with an anti-CD3 bead (Fig. 2Bvii).

### 3.3 SKAP1 deficiency alters anti-CD3 induced talin cleavage

**Fig. 3:**
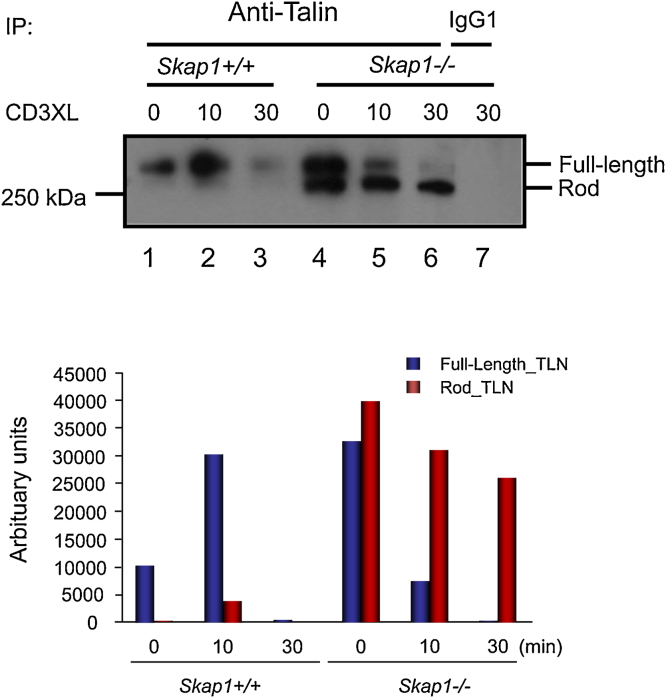
Skap1*−*/*−* T-cells show alterations in anti-CD3 induced talin cleavage. (A) Naïve CD3^+^ T-cells from *Skap1+/+* or *Skap1*−*/−* mice were activating with cross-linking anti-hamster antibodies alone (0) or with anti-CD3 cross-linked with anti-hamster antibodies for either 10 min (10) or 30 min (30). The cytoplasmic and membrane fractions were isolated as indicated and immunoprecipitated with anti-talin. A negative control sample immunoprecipitated with IgG1 antibodies was included (lane 7). The immunoblots were probed with anti-talin to detect the full length (∼235 kDa) or rod-domain of talin (∼180 kDa). (B) The relative densitometric reading of the full length or rod-domain from lanes 1–6 for the membrane fraction were measured using ImageJ.

Given the effect of SKAP1 deficiency on talin recruitment to the IS, we next assessed whether SKAP1 might affect the activation status of talin. Talin is cleaved by calpain in a process needed for the disassembly of the focal adhesion complex [44]. Anti-talin antibody [8D_4_] recognises intact talin molecule (225 kDa) as well as the proteolytic calpain–cleaved fragment at 190 kDa [55]. Anti-CD3 ligation resulted in the degradation of talin between 10 and 30 min in membranes from *Skap1+/+* T-cells (Fig. 3, lanes 2,3) [55]. None of the lower Mr proteolytic fragment was found associated with membranes. By contrast, talin and the calpain fragment was observed in membranes of resting *skap1*−*/*−** T-cells (lane 4). Further, full-length talin was more quickly cleaved from 0 to 10 min with the appearance of the cleaved fragment (lane 4–6) (lower histogram). This observation showed that talin was more readily cleaved in the absence of SKAP1 with the accompanying presence of the cleaved fragment of talin with the membranes of resting and anti-CD3 activated cells.

### 3.4 Non-cleavable talin L432G rescued the conjugation defect of SKOT1CTLs

**Fig. 4:**
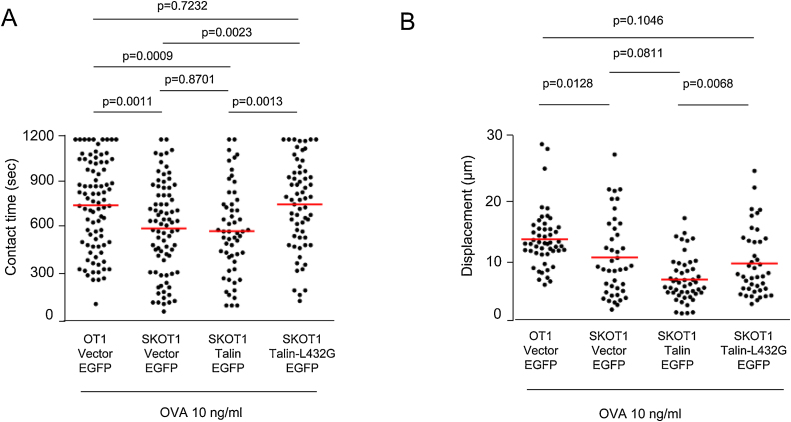
Non-cleavable talin L432G rescued the conjugation defect of SKOT1CTLs. CTLs were transfected with either empty EGFP (EGFP), talin-EGFP or talin-L432G-EGFP vectors. (A) In the presence of DC-OVA, SKOT1CTLs transfected with EGFP ([SKOT1 vector EGFP] mean ± SD, 610.0 ± 33.7 s) showed significant decrease in contact time as compared with OT1 transfected with EGFP. (B) Displacement of SKOT1 CTLs transfected with EGFP were significantly lower in contrast to OT1 CTLs transfected with EGFP in the presence of DC-OVA (*p* < 0.05). The displacement of SKOT1 CTLs transfected with talin-EGFP was comparable to those transfected with EGFP alone (*p* > 0.05). Transfection of Talin-L432G into SKOT1 cells restored displacement to levels seen with OT1 cell transfected with EGFP alone in presence of DC-OVA.

Given this result, we next postulated that a calpain resistant form of talin (L432G-EGFP) might rescue the defect in APC conjugation (Fig. 4). For this, *Skap1*−*/*−** OT 1 T-cells were transfected with either empty EGFP (EGFP), talin-EGFP or talin-L432G-EGFP followed by an assessment of conjugation. Green cells expressing EGFP and their behaviour were visualized and scored under the confocal microscope. *Skap1+/+* OT-1 (OT1) vs*. Skap1*−*/*−** OT-1 (SKOT1) were activated for 3 days with 10 μg/ml OVA peptide, washed and rested for 24 h followed by a measure of dwell times with DCs and motility (Fig. 4). No difference in the expression of LFA-1 was observed on the cells. Mature DCs were prepared as described previously by labeling with SNARF-1 [51]. In the presence of OVA peptide, as expected, OT1 cells showed longer contact times with APCs as compared to SKOT1 cells (a mean of 765 s [OT1 EGFP] relative to 610 s [SKOT1 EGFP] (*p* value = 0.001) (Fig. 4A). The expression of talin-EGFP in SKOT1 T-cells was unable to increase the contact time with DC-OVA. However, the expression of the L432G mutant significantly increased contact times of SKOT1 cells (a mean of 770 s [SKOT1 talin-L432G EGFP] vs. 594 s [SKOT1 talin EGFP]) (Fig. 4A). The mean contact of SKOT1 expressing talin-L432G EGFP was comparable to wild-type OT1 T-cells expressing vector-EGFP. L432G-EGFP also increased displacement of SKOT1 cells (Fig. 4B). These data demonstrated that the reduced conjugation of *Skap1*−*/*−** T-cells could be rescued by the expression of a form of talin that is resistant to cleavage.

In summary, our findings show that the interface localization and processing of talin is regulated by SKAP1 and that the expression of a cleavage resistant talin (L432G) reversed the reduced conjugation of *skap1−/−* OT-1 T-cells with DCs. *Skap1*−*/*−** OT-1 T-cells also showed reduced talin localization to the T-cell interface with antigenic surfaces and DCs and enhanced talin cleavage in resting and anti-CD3 activated T-cells. Talin is necessary for F-actin polarization, the stability of the IS and sustained T cell–APC interactions [56]. Conversely, talin cleavage is needed for the disassembly of the focal adhesion complex [44]. Talin processing may therefore be needed for the disassembly of the contact interface between T-cells and antigen-presenting cells. The enhanced talin cleavage of *Skap1*−*/*−** cells might involve a more rapid disassembly of the contact between T-cells and APCs. Expression of non-cleavable talin might therefore stabilize the contact interface and increase dwell times. The SKAP1 pathway also modulates the transport of RapL to Rap1 in binding to the αL chain of the LFA-1 receptor [23], [24].

T-cell interaction with APCs involves the formation of the central supramolecular activation complex (SMAC [cSMAC]) and peripheral SMAC (pSMAC) in a variety of different cell types [57]. The c-SMAC includes molecules such as CD2, CD28, PKC-θ, Lck, Fyn, CD4, and CD8, while talin and LFA-1 reside in the p-SMAC [58]. Consistent with this, we observed talin to be localized in outer contact regions with anti-CD3 on beads. The loss of SKAP1 in *skap1*−*/*−** primary T-cells resulted in a significant reduction on the localization of talin and RIAM in this outer region and in the case of interactions between T-cells and antigen-presenting cells showed differences in the degree of polarization and length of cell extension. Whether this altered localization of talin was responsible for the reduce localization and increased polarization and size of contact or vice versa is unclear. This reduction in levels of localized talin at the activation interface could predispose *Skap1*−*/−* T-cells to have less stable conjugation due to deficiency of the scaffold for cytoskeletal reorganization [12]. Talin interacts with paxillin, vinculin and zyxin of the cytoskeleton [44]. The importance of full-length talin in conjugation was demonstrated by the ability of protease resistant talin-L432G to restore the dwell times of SKOT1 cells to that of OT1 cells. Consistent with this, a previous report showed that full-length talin rescued adhesion defects of *Talin1*−*/−* T-cells [40]. Overall, our findings indicate that SKAP1 regulates both the affinity and avidity of integrins [23], [24] leading to altered conjugation with the activating interface of anti-CD3 beads or cells.

The SKAP1-talin connection may interface with other adhesion pathways. Talin has also been reported to act downstream of Rap1A, as constitutively active Rap1A (G12 V) failed to activate αIIbβ3-integrin in cells expressing low levels of talin [59]. Rap1-interacting adaptor molecule RIAM has also been implicated in talin activation in the absence of Rap1A (G12 V) (22). Calpain-specific inhibitors block T-cell proliferation and cell shape changes upon TCR activation [55]. Calpain I is sensitive to micromolar levels of Ca2^+^, in comparison to calpain II which requires millimolar levels of Ca^2+^ [60], [61]. It is possible that calpain I, the more sensitive enzyme to Ca^2+^ present in the cytoplasm after T-cell activation in *Skap1*−*/−* T-cells is freely accessible to the talin present in the cytoplasm, resulting in the pronounced cleavage of talin. Overall, our findings suggest that an interplay between two sets of molecules that bind to different chains of LFA-1 in regulating adhesion, and that the altered cleavage and translocation of talin to the IS may be play a role in the reduced ability of *Skap1*−*/*−** T-cells to form stable conjugates with APCs.
